# Histological changes in testes of rats treated with testosterone, nandrolone, and stanozolol

**Published:** 2013-08

**Authors:** Siti Syairah Mohd Mutalip, Gurmeet Kaur Surindar Singh, Aishah Mohd Shah, Mashani Mohamad, Vasudevan Mani, Siti Nooraishah Hussin

**Affiliations:** *Faculty of Pharmacy, Universiti Teknologi MARA, Puncak Alam Campus, Bandar Puncak Alam, Selangor, Malaysia.*

**Keywords:** *Testosterone*, *Nandrolone*, *Stanozolol*, *Testes*, *Substanceabuse*

## Abstract

**Background:** Anabolic androgenic steroids (AAS) is being used in medical treatments, but AAS also was identified to have the risks of adverse effects towards patients and consumers health.

**Objective:** Present study was conducted to observe the effects of testosterone, nandrolone, and stanozolol (forms of AAS) intake during onset of puberty on the rat testicular histology.

**Materials and Methods:** Juvenile male Sprague-Dawley (SD) rats (n=42) were divided into seven groups and were injected subcutaneously with medium dose of polyethylene glycol-200 (PEG-200) (control), testosterone, nandrolone, and stanozolol for six weeks (PND 41-87). The animals were weighed daily and sacrificed on PND 88. Testes were removed, weighed, and prepared for histological assessment and finally specimens were observed under microscope.

**Results:** The results showed an insignificant increase in mean daily body weight with highest and lowest body weight gained was of 177.6±1.69 gr and 140.0±12.26 gr respectively. There was significant decrease in the testes absolute weight (p≤0.01) in all experimental groups except in the nandrolone 2.5 mg/kg/week treated group. Testicular histology of rats treated with AAS also showed slight changes in the uniformity of arrangements of seminiferous tubules.

**Conclusion:** Data from present study suggests that AAS have been initiating the adverse effects on testicular normal functions in rats during onset of puberty.

## Introduction

Anabolic androgenic steroids (AAS) are synthetic substances derived from modified testosterone molecule, the major natural androgenic and anabolic steroid formed in the interstitial Leydig cells of the testes (1). AAS normally used in medical treatment to complement two separated conditions, first it is used in androgen replacement therapy*, *which normally implemented on patients with androgen deficiency due to hypothalamus, pituitary or testicular genetic disorders. 

Second, AAS also commonly being applied as pharmacological androgen therapy (PAT) in patients with non-androgen-deficient with chronic diseases to improve the quality of life by achieving optimum testosterone effects (2). Although been used in clinical treatments, AAS also been identified to give some adverse effects towards patients and consumers health. This is supported by studies revealing that those promising anabolic effects come together with numerous physical and physiological side effects (3, 4). The reported side effects include acne, testicular atrophy, gynaecomastia and hypertension, arrhythmia and myocardial infarction, depression, increment in RBC cells, impaired diastolic function and decrease in sperm count and mortality (5-8). 

Meanwhile, reproductive effects include reduced libido and sexual impotence, impaired spermatogenesis, prostate hypertrophy in men, and hirsutism, voice deepening and menstrual disturbances in women (3, 4). In line with that, the present study was designed to investigate and determine the effects on the testes morphology and histology following AAS (testosterone, nandrolone and stanozolol) exposure during early pubertal period (onset of puberty). 

## Materials and methods

**Ethical approval**

This experimental study was approved by the Ethical Committee of Research Management Institute (RMI) of University Teknologi MARA (UiTM), Shah Alam, Selangor, Malaysia. 

**Animal care**

Fourty two healthy juvenile male Sprague-Dawley (SD) rats were obtained at postnatal day (PND) 35 [from Genetic Improvement and Farm Technologies (GIFT) Sdn Bhd, Petaling Jaya, Selangor, Malaysia]. The animals were acclimatized for a week and maintained at controlled temperature and humidity (24^o^C, 12-h light/12-h dark cycle) with food and water available ad libitum. Experimental animals were given AAS regimen beginning at the onset of puberty (PND 41), for 5 days/week (until PND 87) and finally were sacrificed. 

**Animal treatment**

All 42 juvenile male SD rats were randomly divided into seven groups with six rats per group. To study the effects of different AAS doses on testes, those groups were assigned according to the following treatment: group 1 serves as control group and received PEG 200 (Merck Schuchardt, Hohenbrunn, Germany), group 2, and group 3 treated with testosterone (Steraloids Inc. Newport, USA) of 2.5mg/kg/week (T2.5) and 5mg/kg/week (T5.0) respectively. Group 4 and group 5 consumed nandrolone (Sigma-Aldrich® St. Louis, MO, USA) of 2.5 mg/kg/week (N2.5) and 5 mg/ kg/week (N5.0) each, finally group 6 and group 7 were given stanozolol (Steraloids Inc. Newport, USA) with dose of 2.5mg/kg/week (S2.5) and 5 mg/kg/week each (S5.0). Treatment were conducted by subcutaneous injection from PND 41 to PND 87 with 5 days/week for six weeks. 

Daily body weight were recorded before treatment for 5 days/week. The AAS dose of 5mg/kg/week was chosen base on the work done by Clark and Fast in 1996 which administered a medium dose in rats (equivalent to the human abuse level on a milligram per kilogram of body weight) (9). 

**Organ weight measurement and histological analysis**

Following treatment on PND88, all experimental animals were sacrificed and testes were removed immediately and measured for the recording of absolute organ weight (AOW), length and width before being processed for histological analysis. 

**Histological analysis**

Measured testes were immediately immersed in Bouin’s solution for fixation and processed until embedded in paraffin for histological analysis. Five micron thick sections were prepared using microtome (microTec Laborgerate GmbH Rudolf-Diesel-Straβe, Walldorf, Germany) and stained using Hematoxylin and Eosin (H&E) method. The specimens were examined under Olympus/3H light microscope-Japan.

**Statistical analysis**

Statistical Package of Social Science (SPPS) version 17.0 was used for data management and analyses. Obtained data were statistically analyzed by student *t*-Test and ANOVA to compare the mean of studied parameters in all subject groups. The value of p<0.05 is considered as significant. 

## Results

**Body weight gain**

**Figure 1:**
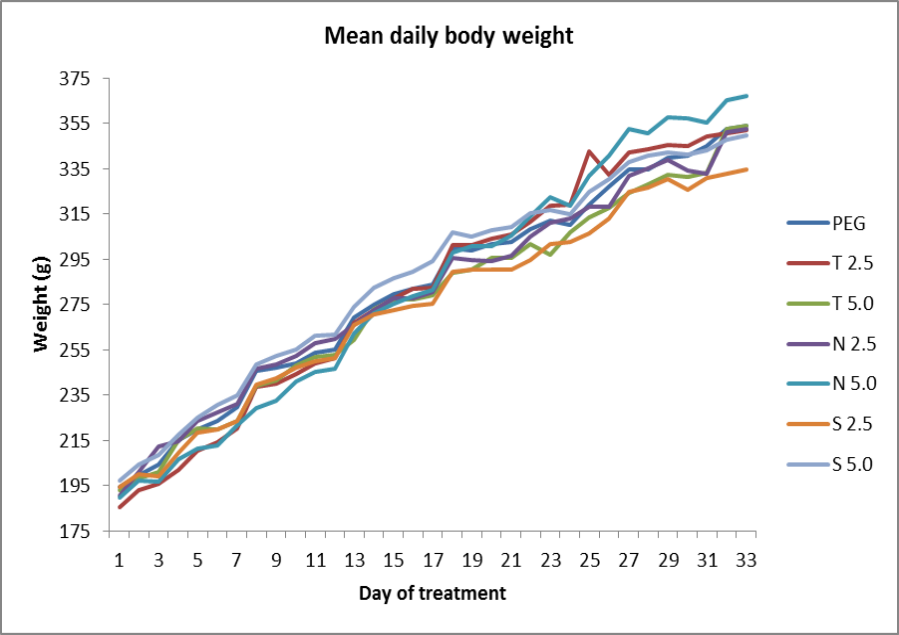
Trend of body weight (g) gain of experimental groups. Nett body weight increment in each experimental group is as follows; control PEG (159.5±8.35 gr), testosterone 2.5mg/kg (T2.5) (166.9±9.85 gr), testosterone 5mg/kg (T5.0) (161.3±7.03 gr), nandrolone 2.5mg/kg (N2.5) (161.9±13.5 gr), nandrolone 5mg/kg (N5.0) (177.6±1.69 gr), stanozolol 2.5mg/kg (S2.5) (140.0±12.26 gr) and stanozolol 5mg/kg (S5.0) (152.5±10.79 gr).

Following six weeks treatment from PND 41 to PND 88, obtained data of SD rats from experimental groups indicates an insignificant increasing pattern in mean daily body weight (Figure 1). Initially they were weighing between 180-200g, but it is found that there are slight enhancement in the body weight measures towards the end of the experiment, with the highest increment was observed in N5.0 group with addition of 177.6 gr and the least body weight gained was from group S2.5 with increment of only 140.0 gr. 

**Absolute testes weight**

**Figure 2:**
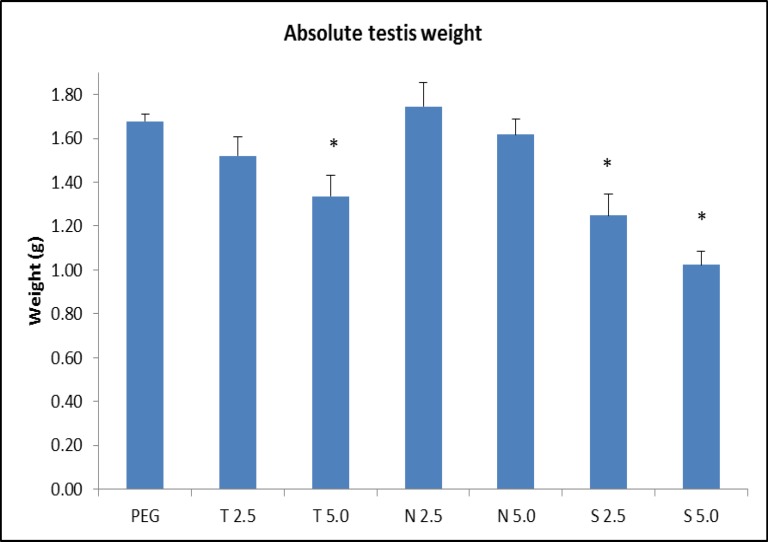
Slight deterioration in testicular size in all treatment groups compared to control group, except for group N2.5 which gives result in slight size increment. Off all these groups, only three groups (T5.0, S2.5 and S5.0) show significant (*) reduction in testicular weight

Obtained data clearly indicates a pattern of decrement in the testicular weight following treatment with testosterone, nandrolone and stanozolol with respective doses. As shown in table I, statistical analysis shows that there are slight deterioration in testicular size in all groups in comparison to control group except for group treated with 2.5 mg/kg/week of nandrolone, which gives result in slight size increment. Off all these testes from AAS-treated SD rats, only three groups (T5.0, S2.5 and S5.0) shows significant reduction (Figure 2). 

**Histological analysis of testes**

Data obtained in the analysis of testicular histology of rats from the control and experimental groups showed a range of differences in the size, shape and uniformity in tubular arrangement. Result of testes histology in control group showed normal and undisturbed pattern in the arrangement and shape of seminiferous tubules with overall mean tubular diameter of 1933.4±120.2 µm, while histological analysis done on the testosterone treated group resulted in slight changes involving the non-intact arrangements of seminiferous tubules, thus leading to development of a wide space between the tubules, besides the shape of the tubules change to become oval-like shape. This changes in the tubular shape results in an elongation of the tubules thus increase the mean diameter of seminiferous tubules with the value of 2142.3±151.7µm in comparison with those of control group. 

As seen from the obtained results, significant histological changes in testes of nandrolone treated group in comparison with testosterone treated group were found in the differences of tubular shapes. The seminiferous tubules in testes from this nandrolone treated group were observed to remain in circular form, just as observed in control group, but with slight reduction in the mean tubular diameter of 1766.3±207.8µm. However, the arrangements of seminiferous tubules are observed to be similar in both testosterone and nandrolone treated groups where both exhibit the appearance of wide interstitial spaces. 

Observed result of testicular tissues from stanozolol treated group clearly depicts shrinkage in overall tubular size with disappearance of tubular lumen. This results in the development of wide interstitial space as observed in both testosterone and nandrolone treated groups. Mean tubular diameter also decreased to 1347.2±176.3 µm compared to control group. However, despite loosening between the arrangements of seminiferous tubules, the overall tubular placement is well in place as what is observed in result from control group. 

Besides differences in testicular morphology, changes were also noticed in the cellular development within the seminiferous tubules. Result from control group shows a normal spermatogenesis cycle with even distribution of every cell stage throughout the tubules. Spermatogonia continue to grow into mature spermatozoa in all tubules. Meanwhile, in testes from testosterone and nandrolone treated group, it were seen that spermatogenesis did took place in all tubules, but in reduced efficacy. Overall amount and distribution of spermatogonia were decreased in nandrolone treated group in comparison to testosterone treated and control group, resulting in lower sperm production. 

In addition, testes from stanozolol treated group exhibits severe reduction in sperm production compare to other treatment groups because spermatogenesis was affected by shrinkage in tubular size resulting in less spermatogonial cells available for development. These adverse effects in testicular cell development with reduction in total sperm production rate especially in nandrolone and stanozolol treated group might be an effect of these AAS.

**Table I:** Average testis weight (AOW) in all treatment groups

**Groups (Treatments)**	**Weight (gr)**	**p-Value**
PEG-200 (Control)	1.6783+0.0340	-
Testosterone 2.5mg/kg (T2.5)	1.5212+0.0861	0.237
Testosterone 5mg/kg (T5.0)	1.3355+0.0944	*0.012
Nandrolone 2.5mg/kg (N2.5)	1.7452+0.1112	0.612
Nandrolone 5mg/kg (N5.0)	1.6182+0.0720	0.648
Stanozolol 2.5mg/kg (S2.5)	1.2485+0.0954	*0.002
Stanozolol 5mg/kg (S5.0)	1.0227+0.0623	*0.000

* p-value is from comparison to control, p<0.05=significant

## Discussion

Looking as an overview at the obtained data from present study, these results suggest that the juvenile reproductive system during the pre-puberty period of treatment has begun to get influenced by exposure of AAS. This is because there are signs of obstructive adverse effects found in testes of experimental rats, thus providing supportive findings from this study. Referring back to the acquired data from present experiment, it clearly depicts that AAS might have been initiating the effects of AAS as early as during onset of pubertal with changes in the normal state of testicular morphology. 

Small increment in body weight and slight deterioration in testes weight following exposure of AAS in all treatment groups is a form of normal effect that would have been expected, and it is observed in this study, as previously reported (10). Significant reduction in testes weight were observed descending in group treated with stanozolol, testosterone and finally nandrolone. This differences in size decrement between treatment groups is basically depends on the molecular structure of the synthetic drugs (11, 12).

In this case, stanozolol has the highest reducing effects on the testicular weight. This is possible because stanozolol is believed to possess anabolic effects which predominate over its androgen effects, perhaps this might be due to the low affinity of stanozolol towards the androgen receptor (13). The high anabolic against androgenic property of stanozolol also results in the least increment in body weight gain (Figure 1), confirming the claim that stanozolol still involves in masculinization (anabolic effects) even at low doses. 

Meanwhile, histological analysis of testes from rats treated with testosterone, nandrolone and stanozolol show effects on seminiferous tubules including differences in normal size and shape where this will definitely results in abnormality of normal testicular functions. For instance, size reduction and shape changes of the tubular does affect the smoothness of spermatogenesis in all treatment groups including delayed and severe reduction in sperm production. These effects are actually resulting from the disturbance in total appearance and distribution of normal spermatogonial cells, which were found to vary within the tubular among treated groups. 

Besides, decrement in tubular size leads to an opportunity whereby the placements of the tubules are seen to be disaggregated and moving away from each other, leaving to development of wide spatial interstitial area between the tubules. This condition, in term of long-term effects, probably has effects on the function of Leydig cells where disturbance in Leydig cells will further affect the normal secretion of testicular testosterone and finally disrupts the entire testicular functions. 

Thus, present result suggest that early exposure to AAS starting from onset of puberty does have effects on the interruption in normal testosterone production which will cause disturbances in regulation of spermatogenesis and eventually leads to decrease in sperm count and mortality as previously reported (7-8, 14). These effects probably will be permanent if AAS is taken continuously as well as there are risks of experiencing decrease in libido and reduction of testicular size after two to three weeks of AAS consumption depending on the type of drug taken as reported before (15, 16). 

## Conclusion

Present study clearly exhibits that AAS has started its effects on the normal state of testes in juvenile rats, including possibilities of having chronic testicular destruction which may lead to male infertility. This is actually an indicator that AAS does have adverse effects to the consumers, regardless the age of first consumption. 

## Conflict of interest

There is no conflict of interest in this work.
